# 1-Methylcyclopropene Alleviates Postharvest Chilling Injury of Snap Beans by Enhancing Antioxidant Defense System

**DOI:** 10.17113/ftb.61.03.23.7860

**Published:** 2023-09

**Authors:** Na Lv, Cai-Ping Wang, Hong-Tao Zhou, Chang-Jie Guo, Hao-Yan Zhang, Da-Yong Ren

**Affiliations:** College of Food Science and Engineering, Jilin Agricultural University, Changchun, 130118 Jilin, PR China

**Keywords:** snap bean (*Phaseolus vulgaris* L.), chilling injury, 1-methylcyclopropene, antioxidant systems, phenolic compounds

## Abstract

**Research background:**

Chilling injury is a major disorder affecting the quality of tropical and subtropical vegetables during low temperature storage. Snap bean (*Phaseolus vulgaris* L.) is sensitive to chilling injury. The main purpose of the present study is to investigate the alleviating effects of 1-methylcyclopropene (1-MCP) on chilling injury of snap bean. In addition, the related mechanisms were also detected from the perspective of the changes of antioxidant defense system.

**Experimental approach:**

Snap beans were exposed to different volume fractions of 1-MCP. After 24 h of treatment, snap beans were stored at 4 °C for up to 14 days. Chilling injury index, electrolyte leakage, titratable acidity and total soluble solids were determined. Contents of chlorophyll, ascorbic acid and malondialdehyde were assessed. The total antioxidant capacity, Fe(II) ion chelating capacity, scavenging capacities on free radicals and activities of antioxidant enzymes were detected. Total phenol content and activities of related metabolic enzymes were also determined.

**Results and conclusions:**

1-MCP treatment reduced chilling injury index, electrolyte leakage rate and malondialdehyde content of snap beans. The amounts of total soluble solids, titratable acid, ascorbic acid and total chlorophyll in 1-MCP-treated snap beans were significantly higher than those of control. The snap beans treated with 1-MCP showed stronger total antioxidant capacity and metal chelating activity. The 1-MCP treatment enhanced scavenging effects of snap beans on superoxide, hydroxyl and 1,1-diphenyl-2-trinitrophenylhydrazine radicals. The activities of peroxidase, ascorbate peroxidase, superoxide dismutase and catalase in 1-MCP-treated group were higher than of control. The treatment also enhanced the accumulation of phenolic compounds in snap beans by regulating the activities of phenol-metabolizing enzymes such as shikimate dehydrogenase, phenylalanine ammonia lyase enzyme, cinnamic acid 4-hydroxylase and polyphenol oxidase. In conclusion, with the mechanism that involves the activation of enzymatic and non-enzymatic antioxidant systems, 1-MCP has the ability to avoid chilling injury of snap bean.

**Novelty and scientific contribution:**

This study gives insights into whether 1-MCP can regulate postharvest cold resistance in vegetables by enhancing the enzymatic antioxidant system and inducing the accumulation of non-enzymatic antioxidants. Considering the results, 1-MCP treatment could be an effective method to alleviate postharvest chilling injury of snap beans during low temperature storage.

## INTRODUCTION

Temperature is an important environmental factor affecting metabolic process, quality and storage period of fruits and vegetables. In general, most fruits and vegetables should be stored at low temperatures after harvest, as low temperatures reduce the respiration of fruits and vegetables. However, many tropical or subtropical vegetables are sensitive to low temperatures. They are vulnerable to chilling injury when stored in low-temperature environment above 0 °C. As a chilling-sensitive vegetable, snap bean (*Phaseolus vulgaris* L.) is prone to chilling injury under low temperature stress for a long time. Therefore, its quality control is needed. Chilling injury in snap bean is characterized by rusty spots on the surface, dark watery patches and discolouration (*1*). In the last few years, many studies have been devoted to looking for ways to control chilling injury.

The relationship between reactive oxygen species (ROS) amount and chilling injury has been widely investigated. Environmental stress such as chilling can trigger the generation of ROS and destroy the balance of ROS in plant cells. The accumulation of ROS can damage the integrity of cell membrane. Then the membrane fatty acids undergo lipid peroxidation to form malondialdehyde (MDA) (*2*). Therefore, maintaining intracellular ROS homeostasis is very important for alleviating chilling injury of vegetables during low temperature storage. The intracellular antioxidant system with ROS clearance is divided into non-enzymatic and enzymatic antioxidant systems. The enzymatic antioxidant systems consist of a series of antioxidant enzymes such as catalase (CAT), peroxidase (POD), superoxide dismutase (SOD) and ascorbate peroxidase (APX), which immediately quench the ROS to protect the membrane from oxidative damage. The non-enzymatic antioxidants include ascorbic acid, polyphenols, tocopherol, and so on (*3*).

The 1-methylcyclopropene (1-MCP) is a small cyclic hydrocarbon molecule. It is a type of ethylene receptor inhibitor. With the inactivated receptors, the tissue no longer responds to ethylene even if it is present. Recently, the application of 1-MCP as postharvest treatment has been considered for enhancing the quality of horticultural crops (*4*, *5*). However, there are few studies of the prevention and control of chilling injury of snap beans with 1-MCP during low temperature storage. The main purpose of the present study is to investigate the alleviating effects of 1-MCP on chilling injury of snap beans. In addition, the related mechanisms were also detected from the perspective of the changes of antioxidant defense system.

## MATERIALS AND METHODS

### Chemicals

The chemicals used in this study were of analytical grade. The 1-MCP, pyrogallol, nicotinamide adenine dinucleotide phosphate (NADP), polyvinylpyrrolidone, polyethylene glycol, *trans*-cinnamic acid, trichloroacetic acid, ascorbic acid, 2,6-dichloroindophenol, guaiacol, EDTA and salicylic acid were obtained from Yuanye, Shanghai, PR China. The 1,1-diphenyl-2-trinitrophenylhydrazine radical, pyrogallol, ferrozine, 2,4,6-tripyridin-2-yl-1,3,5-triazine (TPTZ), riboflavin and phosphate buffer were obtained from Aladdin, Shanghai, PR China. Thiobarbituric, gallic and glacial acetic acid, and nitroblue tetrazolium were purchased from Suolaibao, Beijing, PR China.

### Snap beans and their treatment

Snap beans (*Phaseolus vulgaris* L. cv. ‘Jiuyueqing’) were harvested during a typical commercial ripening period from a farm in Changchun, PR China. They were then delivered to the laboratory within 1 h. All samples were uniform in size and colour without mechanical damage. Each treatment had three replicates with 400 snap beans in each replicate. Snap beans of each treatment were placed in a 40-litre sealed container and exposed to 0.5, 1, 1.5, 2 and 2.5 μL/L 1-MCP. Control beans were exposed to air. Mini fan was used to keep the air circulation. After 24 h of treatment, all snap beans were stored at 4 °C and 75 % relative humidity (RH) for up to 14 days. Snap beans were randomly taken at 2-day intervals. Chilling injury index was assessed immediately after sampling at 4 °C. Twenty snap beans were used to determine electrolyte leakage, titratable acidity and the content of malondialdehyde in the pericarp. The remaining beans were immediately frozen in liquid nitrogen and stored at −80 °C for further analysis. All experiments were performed in triplicate.

### Chilling injury index

Chilling injury (CI) of snap beans is characterized by rusty spots on the surface, dark watery patches and discolouration (*1*). The CI grade was arbitrated as follows: 0=no abnormality, 1=small watery patches or rusty spots, no discolouration, 2=moderate watery patches or rusty spots, no discolouration, 3=severe watery patches or rusty spots, slight discolouration, and 4=extremely severe watery patches, large rusty spots, discolouration of the entire pod. CI index was determined by using the following formula:

CI index=∑[(CI_grade_)·(*N*(fruit)_CI grade_)]/(4·*N*(fruit)_treated total_) /1/

### Electrolyte leakage, malondialdehyde, total soluble solids, titratable acidity, chlorophyll and ascorbic acid content

Electrolyte leakage of snap beans was measured as the total conductivity using the method described by Wang *et al*. (*6*). Bean pod plate was made with a 7 mm diameter punch. A test tube was filled with 2 g of bean pods and 20 mL of deionized water. After shaking, conductivity was determined with a conductometer (DDS-11A; Suoshen Co., Shanghai, PR China). Then, the tubes were boiled for 15 min. After cooling down, the total conductivity of the solution was tested again. Thiobarbituric acid reactive substances (TBARS) method was used to measure malondialdehyde (MDA) content (*2*). Results were expressed in micromoles of MDA per kilogram of snap bean pods. Total soluble solids (TSS) mass fraction was measured using refractometer (WYT; Taihua Optical Co., Chengdu, PR China). Titratable acidity was measured by titration (*7*). A mass of 20 g of bean pods was homogenized in 250 mL of distilled water. After centrifugation (Neofuge 23R; Heal Force Instrument Co., LTD, Shanghai, PR China) at 10 000×*g* for 30 min, the supernatant was collected and used to measure the titratable acidity. A volume of 20 mL of supernatant was titrated with 0.01 M NaOH until the colour of the solution changed to pink (phenolphthalein indicator). Results were represented in g of malic acid per 100 g of bean pods. A method described by Hmmam *et al*. (*8*) was used to detect the content of chlorophyll. Under dim light, 5 g of bean pods were ground with 50 mL acetone and 0.1 g CaCO_3_ in a prechilled mortar. After centrifugation (Neofuge 23R; Heal Force Instrument Co., LTD) at 12 000×*g* for 10 min, the supernatant was adjusted to 50 mL with acetone. Absorbance (UV-26001; Shimadzu Scientific Instruments, Suzhou, PR China) was determined at 663 nm (chlorophyll a) and 645 nm (chlorophyll b), using acetone 95 % as a blank. The 2,6-dichlorophenol indophenol method (*9*) was adopted to analyse ascorbic acid content.

### Total antioxidant capacity, metal chelating activity and free radical scavenging activity

Fe(III) reducing antioxidant power FRAP method was used to detect the total antioxidant activity of snap beans (*10*). A mass of 10 g of bean pods was homogenized with 50 mL of distilled water and centrifuged (Neofuge 23R; Heal Force Instrument Co., LTD) at 10 000×*g* for 20 min. A volume of 3 mL of TPTZ reagent was mixed with 250 μL supernatant. The reaction solution was bathed in water at 37 °C for 10 min. The absorbance (UV-26001; Shimadzu Scientific Instruments) of the solution was measured at 593 nm using spectrophotometer. The standard curve was constructed with FeSO_4_ solution (25–800 μM). FRAP value was expressed in millimoles of Fe(II) equivalents per kilogram of snap beans. The method of Deng *et al*. (*11*) was used to estimate the chelation of Fe(II) ions. The reaction mixture consisted of 1 mL of sample extract, 3.7 mL of ethanol and 0.1 mL of 2 mM solution of FeCl_2_. The reaction was started by adding 0.2 mL of 5 mM ferrozine. Then the mixture was shaken vigorously and kept at room temperature for 10 min. The increase in the absorbance was recorded at 562 nm (UV-26001; Shimadzu Scientific Instruments). Results were expressed as metal chelating activity percentage using the following equation:

Metal chelating activity=((*A*_0_–*A*_1_)/*A*_0_)·100 /2/

where *A*_0_ is the absorbance of the control and *A*_1_ is the absorbance in the presence of the samples.

The experimental procedure described by Zuo *et al*. (*12*) was used to measure the superoxide radical scavenging rate. A mass of 10 g of bean pods was homogenized with 300 mL of distilled water and centrifuged (Neofuge 23R; Heal Force Instrument Co., LTD) at 10 000×*g* for 30 min. To the aliquot of 0.5 mL of supernatant, 4.43 mL of 50 mM Tris-HCl buffer solution (pH=8.2) were added. Then, the mixture was kept at 25 °C for 20 min. Afterwards, 70 μL of 15 mM pyrogallol solution were added. The absorbance was measured at 325 nm. Fenton reaction was used to detect hydroxyl radical scavenging rate. A mass of 5 g of bean pods was homogenized with 10 mL of distilled water. After centrifugation at 10 000×*g* and 4 °C for 30 min, the supernatant was collected. The reaction system consisting of 2 mL of sample extract, 2 mL of 9 mM salicylic acid-ethanol solution, and 1 mL of 9 mM FeSO_4_ solution was kept at 37 °C for 1 h. The reaction was initiated by the addition of 2 mL of 8.8 mM hydrogen peroxide. The absorbance was determined (UV-26001; Shimadzu Scientific Instruments) at 510 nm. The method described by Sridhar and Charles (*13*) was used to measure 1,1-diphenyl-2-trinitrophenylhydrazine radical (DPPH˙) scavenging rate. A mass of 5 g of bean pods was extracted with 10 mL of distilled water and the homogenate was centrifuged (Neofuge 23R; Heal Force Instrument Co., LTD) at 10 000×*g* for 20 min. The reaction mixture contained 2 mL of supernatant and 2 mL of 20 μM DPPH placed in the dark for 30 min. The increase in the absorbance at 517 nm was recorded (UV-260012; Shimadzu Scientific Instruments). Results were expressed as percentage of scavenging activity ( %).

### Antioxidant enzyme activity

A mass of 2.5 g of bean pods from each treatment was homogenized with 10 mL of 0.2 M cold potassium phosphate buffer. After centrifugation (Neofuge 23R; Heal Force Instrument Co., LTD) at 10 000×*g* and 4 °C for 30 min, the obtained supernatant was the crude extract of enzyme. The APX activity was determined according to Sanches *et al*. (*14*) in a 3-mL reaction mixture of 50 mM, pH=7.0, potassium phosphate, 0.1 mM disodium EDTA and 0.3 mM ascorbate, with 0.5 mL of enzyme extract and 0.5 mL of 0.1 mM H_2_O_2_. The increase in the absorbance was recorded (UV-26001; Shimadzu Scientific Instruments) at 290 nm. CAT activity was measured according to the method of Zuo *et al*. (*12*) in a reaction mixture containing 1 mL of distilled water, 1 mL of 0.2 M potassium phosphate buffer, 0.5 mL of enzyme extract and 0.5 mL of 0.1 M H_2_O_2_. The absorbance was measured at 240 nm. The POD activity was determined by a method of Guo *et al*. (*15*) in the reaction mixture containing 3 mL of 25 mM guaiacol solution, 1 mL of 0.5 M H_2_O_2_ and 0.5 mL of enzyme extract. The absorbance was measured (UV-26001; Shimadzu Scientific Instruments) at 470 nm. Nitroblue tetrazolium (NBT) reduction method was used to determine the activity of SOD, as described by Zuo *et al*. (*12*). An enzyme activity unit was expressed as the amount of enzyme required for a 0.01 change in absorbance per minute. Results were expressed as U/g.

### Total phenolic content and enzyme activity associated with phenolic metabolism

For the determination of total phenolic content, 2 g of bean pods were homogenized in 5 mL of methanol. After centrifugation (Neofuge 23R; Heal Force Instrument Co., LTD) at 10 000×*g* for 30 min, the supernatant was collected. A volume of 0.5 mL of supernatant was mixed with 1 mL of Folin-Ciocalteu reagent and 3 mL of 1 M sodium carbonate. Then the total volume of the mixture was adjusted to 10 mL with distilled water. After the mixture had been kept at 25 °C for 1 h, the absorbance was measured (UV-26001; Shimadzu Scientific Instruments) at 760 nm (*16*). The result was expressed as the mass (in mg) of gallic acid equivalents on a fresh mass basis per g of bean pods.

For determination of shikimate dehydrogenase (SKDH), 2 g of bean pods were homogenized in 6 mL of 50 mM potassium phosphate buffer, pH=6.8, then the sample was centrifuged at 10 000×*g* and 4 °C for 30 min. Then reaction 0.2 mL of supernatant was mixed with 1.9 mL of 100 mM Tris-HCl, pH=9.0, 1.45 mL of 2 mM shikimic acid and 1.45 mL of 0.5 mM NADP (*17*). The absorbance was determined (UV-26001; Shimadzu Scientific Instruments) by the reduction of NADP at 340 nm. For determination of phenylalanine ammonia lyase (PAL), 5 g of bean pods were homogenized in 5 mL extraction buffer, containing 4 % polyvinylpyrrolidone, 0.002 M EDTA and 0.005 M β-mercaptoethanol. The mixture was then centrifuged (Neofuge 23R; Heal Force Instrument Co., LTD) at 10 000×*g* and 4 °C for 30 min. The reaction mixture consisting of 0.2 mL of supernatant, 1 mL of 0.6 mM l-phenylalanine and 2 mL of 0.2 M borate buffer (pH=8.8) was kept at 37 °C for 1 h. The increase in the absorbance was measured (UV-26001; Shimadzu Scientific Instruments) at 290 nm. For determination of cinnamate-4-hydroxylase (C4H), 1 g of bean pods was homogenized in 3 mL of 50 mM, pH=8.9, Tris-HCl buffer solution which contained 4 mM magnesium sulfate, 5 mM ascorbic acid, 10 % glycerol and 0.15 % polyvinylpyrrolidone. Then 0.5 mL of the supernatant was mixed with 2.5 mL reaction solution which contained 50 mM, pH=8.9, Tris-HCl buffer, 2 μM NADP and 2 mM *trans*-cinnamic acid. The increase in the absorbance was measured (UV-26001; Shimadzu Scientific Instruments) at 340 nm. For determination of polyphenol oxidase (PPO), 5 g of bean pods were homogenized in 5 mL of precooled extraction buffer containing 1 % TritonX-100, 1 mM polyethylene glycol and 4 % polyvinylpyrrolidone. PPO activity was measured by the method of Wang *et al*. (*18*). The increase in the absorbance was measured (UV-26001; Shimadzu Scientific Instruments) at 420 nm. Activities were expressed on a fresh mass basis as U/g, where U=(0.01 Δ*A*)/min.

### Statistical analysis

All experiments were repeated three times. Data were expressed as mean±standard deviation (S.D.). Data were statistically analysed by analysis of variance (ANOVA) with SPSS statistical software v. 26.0.0 (*19*). Significant differences were calculated with Duncan’s multiple range tests. A probability of p*<*0.05 was considered statistically significant.

## RESULTS AND DISCUSSION

### Sensory quality of snap beans and chilling injury index

**Fig. 1:**
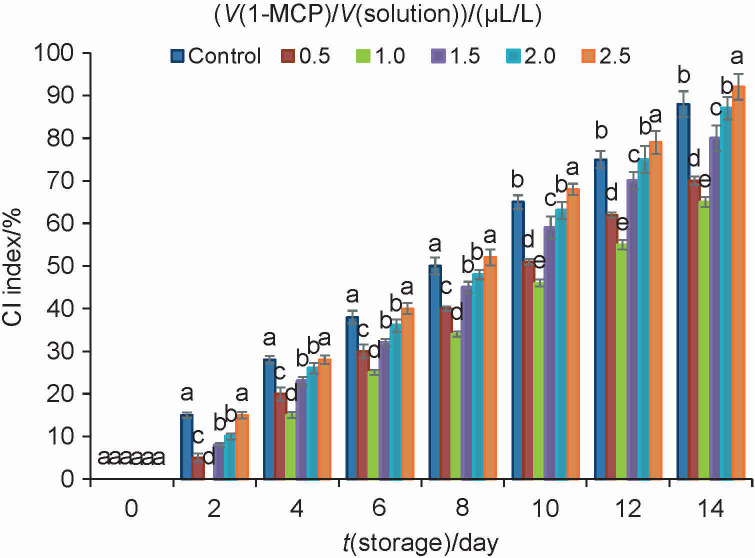
Chilling injury (CI) index of snap beans treated with 1-methylcyclopropene (1-MCP) and control. Snap beans were stored at 4 °C for up to 14 days. Data are presented as mean value±S.D. of three replications. Different letters indicate significant differences among treatments according to Duncan’s test at p=0.05

**Fig. S1:**
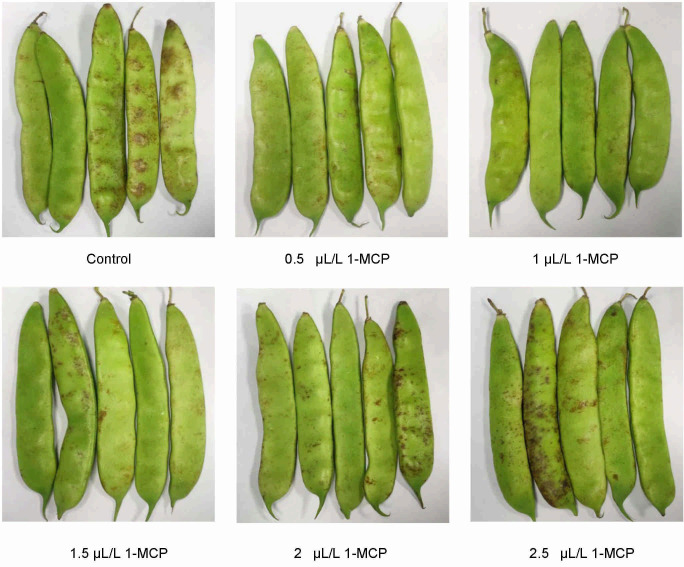
The visual image of chilling injury in snap beans. Snap beans were stored at 4 °C for up to 14 days

The most typical chilling symptoms of snap beans include discolouration, dark watery patches and rusty spots on the surface (*1*). Snap bean is sensitive to chilling temperature and very vulnerable to chilling injury (CI). In fact, the difference in the sensitivity of snap beans to CI significantly depends on the cultivar (*20*). There was no sign of CI on snap beans cv. ‘Opus’ stored at 1 °C. Snap beans of ‘Romano’ cv. were only slightly affected when stored at 5 °C for 2 weeks. Due to chilling injury, postharvest storage time of cultivars ‘Tendergreen’ and ‘Top Crop’ was reduced by 40 %. Chilling injury symptoms of ‘Leon’ snap beans became apparent 2 days after exposure to 1 or 5 °C and 3 days after exposure to 10 °C (*1*). In our study, CI symptoms in control group appeared 2 days after exposure to 4 °C. After 6 days of storage at 4 °C, bean pods showed obvious symptoms of chilling injury. At that time, the sensory quality of snap beans reached the limit of acceptance. Fig. S1 shows the differences in the appearance of snap beans on the 14th day. Bean pods were severely affected and showed many rusty spots and dark watery patches on the surface, especially the control and the group treated with 2.5 μL/L 1-MCP. Fig. 1 shows that the treatment with 1-MCP significantly reduced the CI index of snap beans. The most effective amount of 1-MCP was 1 μL/L. However, CI index of the group treated with 2.5 μL/L 1-MCP was higher than that of control. This indicates that high amount of 1-MCP aggravated chilling damage of snap bean, and led to the appearance of more rusty spots at the end of storage. The 1-MCP can irreversibly bind to ethylene receptors, thereby avoiding subsequent ethylene response. It has been demonstrated that 1-MCP prevents deterioration of quality by delaying senescence, as well as by inducing chilling tolerance in many vegetables. Although postharvest treatment with 1-MCP is effective and nontoxic, its effectiveness is highly variable. This study showed that its effectiveness is directly related to the amount used in the treatment.

### Electrolyte leakage, malondialdehyde, total soluble solids, titratable acidity, chlorophyll and ascorbic acid content

**Fig. 2:**
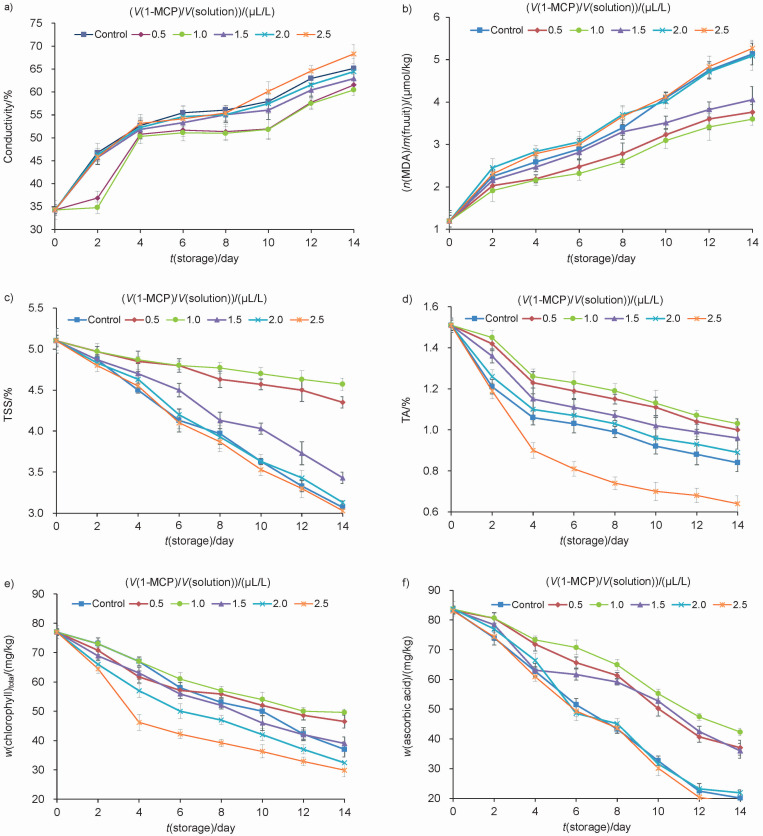
Determination of: a) conductivity, b) malondialdehyde (MDA), c) total soluble solids (TSS), d) titratable acidity (TA), e) chlorophyll and f) ascorbic acid content of snap beans stored at 4 °C for up to 14 days. Data are presented as mean value±S.D. of three replications

Membrane permeability is usually indicated by the change in electrolyte leakage. Electrolyte leakage is a good qualitative index of chilling sensitivity. As shown in Fig. 2a, conductivity of all groups increased with the storage time. In control group it increased from 34.26 to 65.14 %. However, contrary to the control group, the treatment with 1 μL/L 1-MCP delayed electrolyte leakage. Chilling injury can enhance membrane lipid peroxidation and produce MDA (*8*). Oxidative stress in fruits and vegetables can be detected directly as the accumulation of MDA. Fig. 1 and Fig. 2b show that the content of MDA increased with the increase of CI index under low temperature stress, indicating that the chilling stress had exacerbated the degradation of membrane lipids and may lead to the deletion of cell integrity. Compared with the control group, the content of MDA in the group treated with the amount of 1 μL/L 1-MCP was the lowest (Fig. 2b). These results clearly demonstrate that 1-MCP could inhibit the accumulation of MDA and reduce electrolyte leakage, suggesting that the membrane integrity was maintained when exposed to 1 μL/L 1-MCP. Similar results of chilling tolerance induced by 1-MCP were reported in nectarine (*3*) and persimmon (*21*).

Total soluble solids (TSS) and titratable acidity are important quality indices of vegetables (*22*). Chilling injury induced rapid plant senescence and had negative effects on these quality attributes (*23*). As can be seen in Fig. 2c, TSS contents in all groups decreased continuously during refrigerated storage. TSS content in control group decreased from 5.10 to 3.07 %. The treatment with 0.5 and 1 μL/L 1-MCP significantly prevented the decrease of TSS. However, the other groups were not statistically significantly different compared to control (p>0.05). Fig. 2d shows that titratable acidity continuously decreased throughout 14-day storage in all groups. However, the titratable acidity was noticeably higher in groups treated with 0.5 and 1.5 μL/L 1-MCP than in the control. The titratable acidity in the group treated with 1 μL/L 1-MCP was the highest, which may be related to the lowest CI index in this group, thus inhibiting the rapid senescence of snap beans.

Discolouration is one of the most common symptoms of CI observed in snap beans. Chlorophyll is an important factor to determine the acceptability of snap beans by consumers. The chlorophyll degradation usually reflects the quality deterioration of snap beans (*24*). Chlorophyll mass fraction significantly decreased as the colour of the snap bean pods turned from a bright green to a more yellowish green. Fig. 2e shows that the total mass fraction of chlorophyll on fresh mass basis decreased from 77.01 to 32.20 mg/kg in the control group. However, treatment with 1 μL/L 1-MCP delayed the chlorophyll degradation. Ascorbic acid is often considered as an important non-enzymatic antioxidant bioactive compound which scavenges ROS (*25*). The decrease of ascorbic acid amount is usually associated with the ageing process in plants. Fig. 2f shows that the mass fraction of ascorbic acid in snap beans decreased continuously during storage. Treatment with 1 μL/L 1-MCP significantly prevented the decrease of ascorbic acid.

The above results suggested that the optimal amount of 1-MCP used for cold storage of snap beans was 1 μL/L and consequently it was used for the next experiments.

### Total antioxidant capacity, metal chelating activity and free radical scavenging rates

**Fig. 3:**
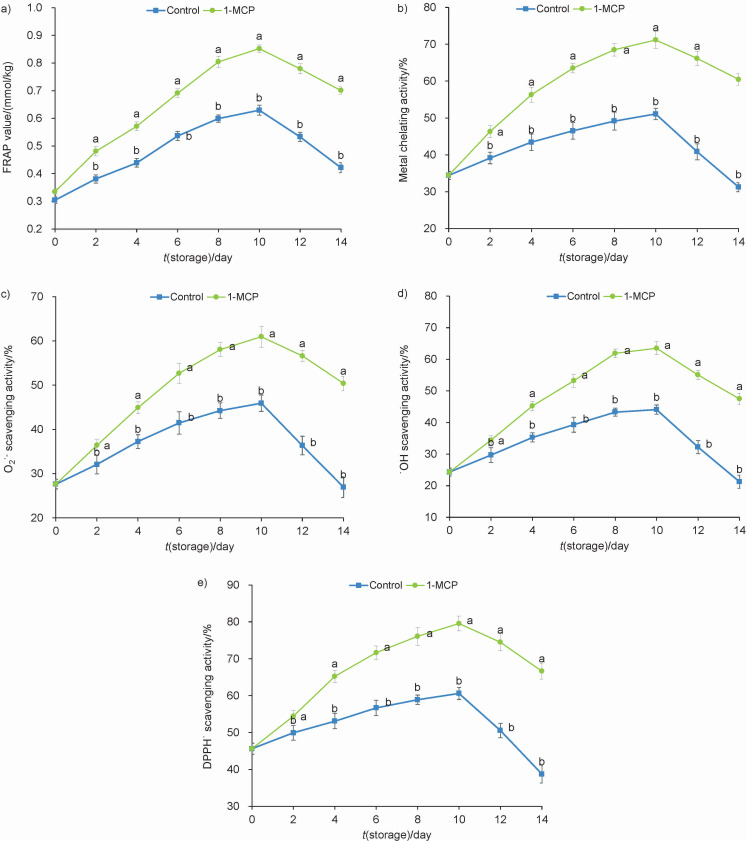
Determination of: a) total antioxidant capacity, b) metal chelating activity, and free radicals: c) O_2_˙^-^, d) ˙OH and e) DPPH˙ scavenging activity of snap beans stored at 4 °C for up to 14 days. Data are presented as mean value±S.D. of three replications. Different letters indicate significant differences among treatments according to Duncan’s test at p=0.05. *V*(1-MCP)/*V*(solution)=1 μL/L

Results in Fig. 3a show that the FRAP value increased initially and then decreased. At the end of storage, a maximal increase of 1.66-fold of the control value was seen in the group treated with 1-MCP. Similarly, as shown in Fig. 3b, compared to untreated control, treatment of snap beans with 1-MCP resulted in 1.93-fold higher metal chelating activity on the 14th day. This result indicates that 1-MCP can inhibit the formation of Fe^2+^-ferrozine complex in snap beans.

ROS such as O_2_˙ and ˙OH are the most prevalent radicals in plant cell. DPPH is widely used in the evaluation of reducing substances. Figs. 3c–3e show that free radical scavenging rate increased during the initial ten days of storage and decreased thereafter. At the end of storage, scavenging rates of O_2_˙^-^, ˙OH and DPPH˙ in control beans were 46.52, 55.30 and 41.86 % lower than those in the beans treated with 1-MCP, respectively. The enhanced tolerance to chilling injury in 1-MCP-treated snap beans was associated with increased levels of free radical scavenging, which could be related to changes in antioxidant enzyme activities.

### Antioxidant enzyme activities

**Fig. 4:**
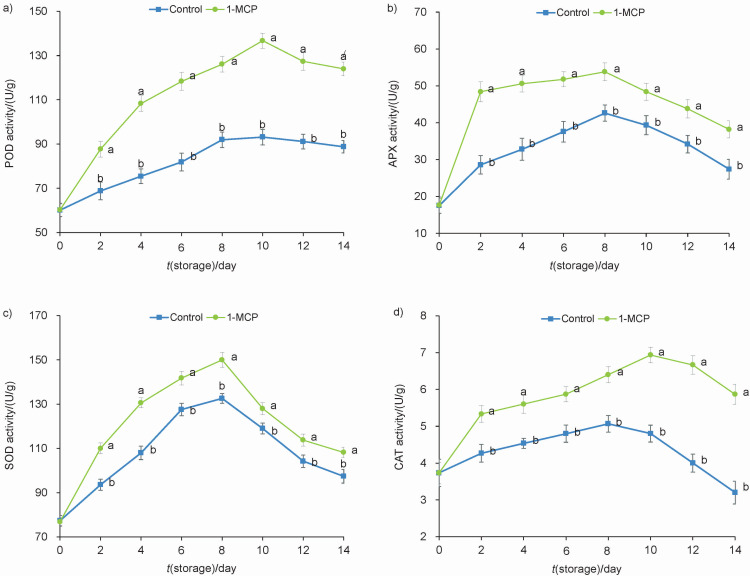
Activities of: a) peroxidase (POD), b) ascorbate peroxidase (APX), c) superoxide dismutase (SOD) and d) catalase (CAT) of snap beans stored at 4 °C for up to 14 days. Data are presented as mean value±S.D. of three replications. Different letters indicate significant differences among treatments according to Duncan’s test at p=0.05. *V*(1-MCP)/*V*(solution)=1 μL/L

It has been reported that the chilling tolerance of vegetables is positively correlated with the activity of antioxidant defence system (*26*). Low temperature stress is also a kind of stress that not only negatively affects the membrane structure of chilling-sensitive vegetables, but also reduces the activity of antioxidant enzymes (*27*). POD, CAT, APX and SOD are important components of the antioxidant system in vegetables and have the ability to remove ROS (*28*). To investigate the effect of 1-MCP on enzymatic antioxidant system of snap beans, activities of POD, APX, SOD and CAT were determined. As shown in Fig. 4, snap beans exposed to 1-MCP showed significantly higher activities of POD, APX, SOD and CAT than control beans. These results clearly indicate that the enhanced antioxidant activity of 1-MCP-treated snap beans was achieved by inducing the activities of POD, APX, SOD and CAT antioxidant enzymes.

### Total phenolic content and activities of SKDH, PAL, C4H and PPO

**Fig. 5:**
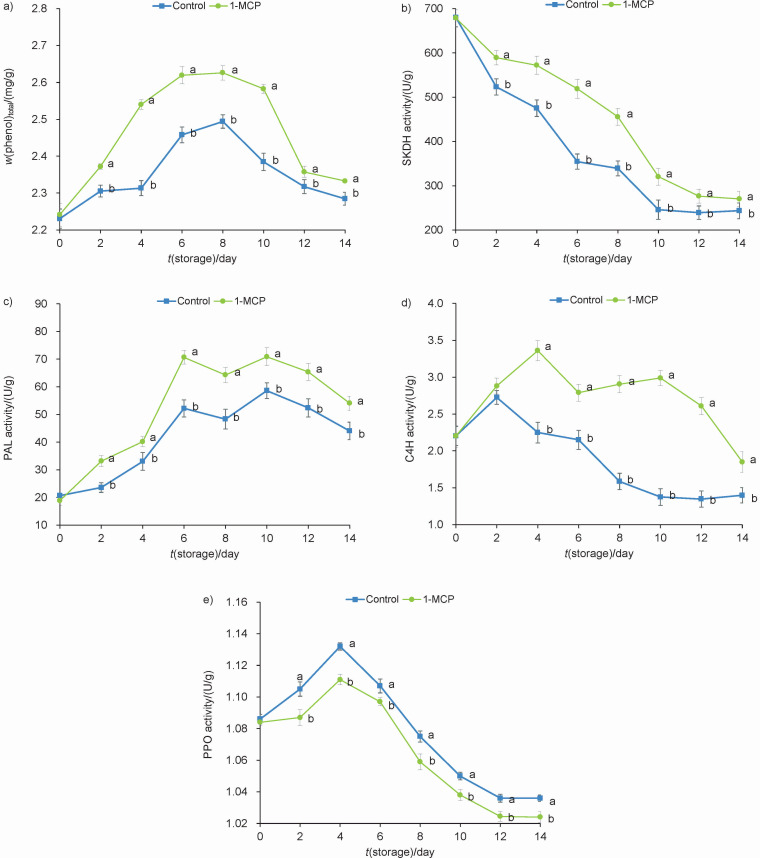
Determination of: a) total phenolic content (TPC) and activities of: b) shikimate dehydrogenase (SKDH), c) phenylalanine ammonia lyase (PAL), d) cinnamate-4-hydroxylase (C4H) and e) polyphenol oxidase (PPO) of snap beans stored at 4 °C for up to 14 days. Data are presented as mean value±S.D. of three replications. Different letters indicate significant differences among treatments according to Duncan’s test at p=0.05. *V*(1-MCP)/*V*(solution)=1 μL/L

Non-enzymatic antioxidants such as phenolic compounds also play an important role in the removal of ROS during stress tolerance (*29*). As shown in Fig. 5a, 1-MCP treatment delayed the decrease of total phenolic content during the whole storage period. During storage from 8 to 14 days, the total phenolic content in control group decreased on fresh mass basis from 2.49 to 2.28 mg/g, and in the group treated with 1-MCP it decreased from 2.63 to 2.34 mg/g. Considering the results, the improvement of free radical scavenging ability could be associated with the increase of total phenolic content, which prevents membrane lipid peroxidation (*30*). Snap beans treated with 1-MCP showed significantly (p<0.05) higher activities of shikimate dehydrogenase (SKDH), phenylalanine ammonia lyase (PAL) and cinnamate-4-hydroxylase (C4H) than control during cold storage (Figs. 5b–5d). Fig. 5e shows that the polyphenol oxidase (PPO) activity of snap beans exposed to 1-MCP was significantly lower than that of control (p<0.05). The metabolism of phenolic compounds is closely related to the activities of SKDH, PAL, C4H and PPO. SKDH is the key enzyme that catalyzes shikimic acid to produce l-phenylalanine. PAL and C4H are key and rate-limiting enzymes for the synthesis of phenolic compounds. PPO plays an important role in the browning of cold-damaged horticultural crops (*31*). In this study, the PPO activity of snap beans treated with 1-MCP was lower, which may be related to the lower intensity of browning and chilling injury. Taken together, our results suggested that 1-MCP treatment increased total phenol content by enhancing the activities of SKDH, PAL and C4H, and inhibited the activity of PPO, which increased the total phenol content in snap beans.

## CONCLUSIONS

Results of this study showed that 1-methylcyclopropene (1-MCP) assisted in avoiding chilling injury of snap beans. Postharvest treatment of snap beans with 1-MCP inhibited the accumulation of malondialdehyde (MDA) and reduced electrolyte leakage. The treatment with 1-MCP caused a decrease in the consumption of organic acids as respiratory substrates. It significantly prevented the loss of total soluble solids and total chlorophyll. The 1-MCP-treated snap beans showed stronger total antioxidant capacity and metal chelating activity. The treatment enhanced the scavenging effects of snap beans against superoxide, hydroxyl and 1,1-diphenyl-2-trinitrophenylhydrazine radicals. Its effectiveness is directly related to the amount used in the treatment. The optimal amount of 1-MCP to avoid chilling injury in snap beans is 1.0 μL/L. The mechanism involved the activation of enzymatic and non-enzymatic antioxidant systems. Treatment with 1-MCP stimulated the activities of ascorbate peroxidase (APX), peroxidase (POD), superoxide dismutase (SOD) and catalase (CAT) in snap beans, which are important enzymes in the enzymatic antioxidant system. Besides, 1-MCP treatment enhanced the accumulation of non-enzymatic antioxidants such as ascorbic acid and phenolic compounds in snap beans. The increase of total phenol content in 1-MCP-treated snap beans was related to the regulation of shikimate dehydrogenase, phenylalanine ammonia lyase enzyme, cinnamic acid-4-hydroxylase and polyphenol oxidase. Accordingly, treatment with 1.0 μL/L 1-MCP is probably a good way to maintain the storage quality of snap beans during low-temperature storage.
